# Living Ring-Opening Polymerization of *O*-Carboxyanhydrides: The Search for Catalysts

**DOI:** 10.3389/fchem.2018.00641

**Published:** 2018-12-21

**Authors:** Yongliang Zhong, Rong Tong

**Affiliations:** Department of Chemical Engineering, Virginia Polytechnic Institute and State University, Blacksburg, VA, United States

**Keywords:** *O*-carboxyahydrides, polyester, ring-opening polymenzation, photoredox catalysis, living polvmerization, organocatalyst, stereoselective polymerization, poly(a-hydroxy acid)

## Abstract

Biodegradable poly(α-hydroxy acids) can be synthesized by means of ring-opening polymerization (ROP) of *O*-carboxyanhydrides (OCAs). Numerous catalysts have been developed to control the living polymerization of OCAs. Here we review the rationale for the use of OCA, the desirable features for and important attributes of catalysts for the ROP of OCAs, and specific examples that have been developed.

## Introduction

Polymers, commonly called plastics, can be categorized as degradable and non-degradable. Non-degradable plastics, mostly from petrochemical resources, tend to have difficulty in recycling and ultimately pollute the environment (Jambeck et al., 2015; Sardon and Dove, 2018). Substantial efforts have been devoted to develop degradable polymers (Zhu et al., 2016). Poly(α-hydroxy acids), including polylactide (PLA) and polyglycolide, and other polyesters are arguably the most successful examples (Middleton and Tipton, 2000; Dechy-Cabaret et al., 2004; Danhier et al., 2012). However, the mechanical and thermal properties of these materials still need to be improved to match non-degradable polymers (Jacobsen et al., 1999; Farah et al., 2016).

**Figure 1:**
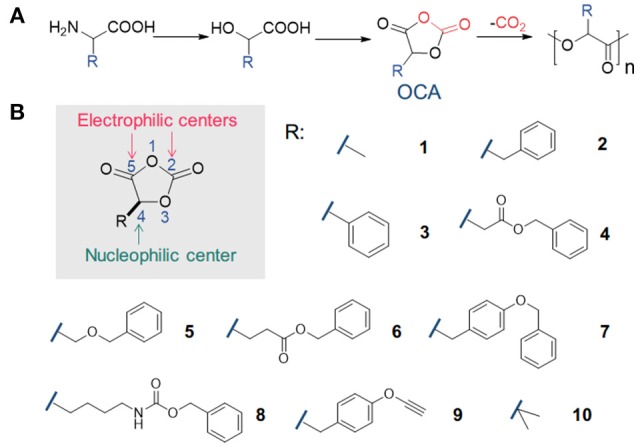
Synthesis and polymerization of *O*-carboxyanhydrides (OCAs). **(A)** Synthesis of OCAs from α-amino acids, and the polymerization of OCAs to prepare poly(α-hydroxy acids). **(B)** The scheme of reactivity sites in OCA, and representative OCA monomers bearing various functional groups.

Besides efforts in processing with additives or developing new processing techniques (Di et al., 2005; Anderson et al., 2008; Lim et al., 2008; Rasal et al., 2010; Armentano et al., 2013; Nofar and Park, 2014; Kühnert et al., 2018), one major focus within polymer chemistry society is to generate new sets of monomers from natural resources to produce new degradable polymers that potentially replace many commodity polymers in the market (Yu et al., 2014; Gregory et al., 2017; Tong, 2017; Becker and Wurm, 2018). Among these new monomers, 1,3-dioxolane-2,4-diones, so-called *O*-carboxyanhydrides (OCAs), have emerged as active monomers for the synthesis of poly(α-hydroxy acids) (du Boullay et al., 2006; Martin Vaca and Bourissou, 2015; Yin et al., 2015). OCAs can be prepared from α-amino acid or α-hydroxy acids with a rich variety of side-chain functionalities (Figure 1; Martin Vaca and Bourissou, 2015; Yin et al., 2015). Note that the functionalization of corresponding lactide monomers often involves more synthetic steps with lower yields; and the polymerization of those functionalized lactide monomers can be difficult to achieve high molecular-weight (MW) polymers (Bourissou et al., 2007; Yu et al., 2014).

OCAs are polymerized via ring-opening polymerization (ROP). This ROP process has been regarded as thermodynamically more favorable than that of lactide due to the liberation of a carbon dioxide molecule during the polymerization process (du Boullay et al., 2006). However, challenges remain in exploring desirable catalysts for controlled ROP of OCAs, especially for potential industrial application. A number of review articles have discussed the development of polymerization of OCAs (Martin Vaca and Bourissou, 2015; Yin et al., 2015; Feng et al., 2017). It is not the intention of this review to attempt another similar comprehensive review of OCA polymerization, but rather to discuss the problems in ROP of OCAs. We shall outline some general considerations about living ROP of OCA, followed by a discussion of the recent literature from a catalyst-development perspective. On occasion, we refer to some polymerization reactions for purely illustrative purposes. Their mention is not an endorsement, nor is omission to be considered as a negative judgment.

## Considerations in OCA Polymerizations

### Living and Controlled Polymerization of OCAs

Living polymerization—that is, all polymer chains grow at the same rate with no irreversible transfer or termination reactions—is central to current polymer chemistry (Szwarc, 1956; Grubbs and Grubbs, 2017). Generally, the rate of initiation (*k*_i_) should be greater than the rate of propagation (*k*_p_); the addition of a monomer to polymer chain ends occurs irreversibly, without chain termination and side reactions; and the breadth of the MW distribution (*-D*) becomes extremely narrow (*-D* approaches 1) (Grubbs and Grubbs, 2017). Living polymerizations can often be distinguished from kinetically-controlled (i.e., free radical) polymerizations by analyzing the evolution of the polymer's MW as a function of time and/or monomer conversion: MW is directly proportional to monomer conversion in living polymerizations since all chain ends are growing at essentially the same rate.

Based on the consensus of the “living and controlled” polymerization, the generic properties of an ideal OCA polymerization catalytic system are summarized as follows: (1) fast and complete initiation; (2) regioselective ring-opening of OCAs; (3) for practical reasons, converting monomers to growing polymer chains quantitatively and rapidly; (4) a linear relationship between the degree of polymerization (DP, typically measured as the number-average MW of the polymer, *M*_n_) and monomer consumption; (5) *- D* < 1.2, which means the polymerization proceeding without an appreciable amount of (intramolecular or intermolecular) chain transfer or premature termination; (6) capable of controlling MW over a wide range (synthesis of high MW polymers). Last, but not least, for future industrial application, the catalyst should show high stability toward moisture and air, and maintain cost-effectiveness.

### Thermodynamics for OCA Polymerization

At first glance, OCA bears multiple possible sites for nucleophilic attack (Figure 1B), similar to its analog NCA (*N*-carboxyanhydrides) molecule. Nevertheless, early studies by Smith and Tighe suggested that OCA is very stable and shows little tendency to polymerize compared with NCA (Smith and Tighe, 1976):the dimethyl-substituted OCA monomer (**10**) had a half-life over 1,000 h in a 90°C nitrobenzene solvent. However, these results remained relatively obscure over years; instead, the liberation of CO_2_ from OCA monomers has been regarded as a considerable driving force for polymerization (du Boullay et al., 2006), in addition to the ring strain as in many other cyclic monomers for ROPs (Saiyasombat et al., 1998; Odian, 2004; Houk et al., 2008).

One calculation showed that the ring-opening of l-**1** is thermodynamically more favorable in terms of Gibbs free energy (Δ*G*° = −14.0 kcal/mol) than that of lactide (1.2 kcal/mol), catalyzed by 4-dimethylaminopyridine (DMAP) and methanol (du Boullay et al., 2006; Bonduelle et al., 2008). However, such a calculation only considers the initiation step, and the results can be complicated when factors such as chain propagation and different catalysts are involved in. For instance, the ROP of l-**1** mediated by DMAP/*neo*-pentanol affords a controlled polymerization at room temperature (*M*_n_ = 62.3 kDa; *-D* = 1.18) (du Boullay et al., 2006), superior to that of lactide by the same catalyst requiring few days in refluxing solvent (Nederberg et al., 2001). However, recent experimental studies on the yttrium complex-mediated ROPs of **1** showed that the Gibbs free energy of activation of l-**1** and l-lactide were essentially the same (16.5 vs. 16.7 kcal/mol, respectively) (Ouyang et al., 2017). The obtained *k*_app_ (*k*_app_, the apparent rate constant) values for both polymerizations were also in the same order of magnitude with <20% difference; though prolonged induction time for the ROP of l-lactide was observed (Ouyang et al., 2017). In addition, in many cases (Breslow et al., 1957; Penczek et al., 1980; Duda et al., 2005), the fulfillment of thermodynamic requirements is a necessary—but not sufficient—prerequisite for a living polymerization to occur. The effects of catalysts can be seen as pivotal to the success of ROPs; performing polymerization kinetic studies is essential for mechanistic studies.

### Molecular Weight of the Polyester

PLAs with *M*_n_ exceeding 100 kDa can be synthesized by Al(O*i*Pr)_3_ or Sn(II) octanoate-based initiating systems (Dubois et al., 1991; Degée et al., 1999; Kowalski et al., 2000a). For example, the use of Sn(OBu)_2_ allows the polymer *M*_n_ over 900 kDa (Kowalski et al., 2000b). However, until recently, most polymers obtained from the ROP of OCAs have relatively low MWs (<50 kDa). Research in PLA shows that the mechanical properties and crystallization behaviors of PLA are dependent on the MW of the polymer (Garlotta, 2001). For instance, the tensile modulus of PLA increases by a factor of 2 when MW is raised from 50 to 100 kDa (Södergård and Stolt, 2002), whereas tensile strengths increase from 15.5 to 150 MPa when MW varies from 50 to 200 kDa (Van de Velde and Kiekens, 2002). Though PLAs used for biomedical applications often present a MW of about 5–30 kDa (Lasprilla et al., 2012), PLA materials for orthopedic and other temporary implants used in bone surgery usually have MWs from 150 to 300 kDa (Slomkowski et al., 2014). Those used to produce packaging materials necessitate high MW PLAs to exhibit decent mechanical properties (Garlotta, 2001; Auras et al., 2004). Therefore, it is critical for chemists to develop catalysts to enable the synthesis of high-MW polyesters.

## OCA Monomer: Synthesis and Purification

In 1951 Davies first reported the synthesis of OCA by reacting α–hydroxy acid with phosgene, similar to NCA synthesis (Davies, 1951). To date, various OCAs have been synthesized (Figure 1B). In general, α-hydroxy acids are carbonylated using phosgene, diphosgene (Toyooka et al., 1989; Tang and Deng, 2002) or triphosgene (He et al., 2013; Chen et al., 2014). In case of the latter two carbonylation agents, activated charcoal is often used to promote the decomposition to phosgene and sometimes a tertiary amine (e.g., *N*-methylmorpholine) is added as an acid scavenger (Kricheldorf and Jont, 1983; Vandenbossche et al., 2010).

In many cases, repetitive crystallization is enough to obtain pure OCA monomers (du Boullay et al., 2006; Yin et al., 2013). However, methods are still needed for preparation of highly functional or low-melting-point OCAs that are difficult to recrystallize. A few reports suggested the use of flash chromatography for some OCA monomers purification (Vandenbossche et al., 2010; Lu Y. et al., 2012). Notably, NCAs can be purified by flash chromatography in anhydrous environments; (Kramer and Deming, 2010) however, the stability of OCAs in the column and the scope of such a method have not been well studied. The reported rapid and facile microflow synthesis of NCAs is also worth experimenting for OCAs (Otake et al., 2018).

## Organocatalyst for OCA Polymerization

In early studies, the use of amines (e.g., pyridine and trimethylamine) for ROP of OCAs failed to initiate controlled polymerizations, with *M*_n_s < 3 kDa (Smith and Tighe, 1981; Kricheldorf and Jont, 1983). Besides, acidic catalysts (e.g., triflic acid) do not work for the ROP of OCAs (Martin Vaca and Bourissou, 2015). In 2006, the Bourissou group started to apply the organocatalysts that had achieved success in the ROP of lactones to OCA polymerization (du Boullay et al., 2006). Over the years, both DMAP and *N*-heterocyclic carbenes (NHCs) have been utilized for the ROP of OCAs (**1**, **2**, **6**, **8**) and obtained reasonable results (du Boullay et al., 2006, 2008; Lu Y. et al., 2012; Zhang et al., 2012; Chen et al., 2014; Xia et al., 2014). However, most polymers catalyzed by organocatalysts exhibited MWs <30 kDa or low DPs (≤200) (Martin Vaca and Bourissou, 2015).

### The Epimerization of α-Proton

**Figure 2:**
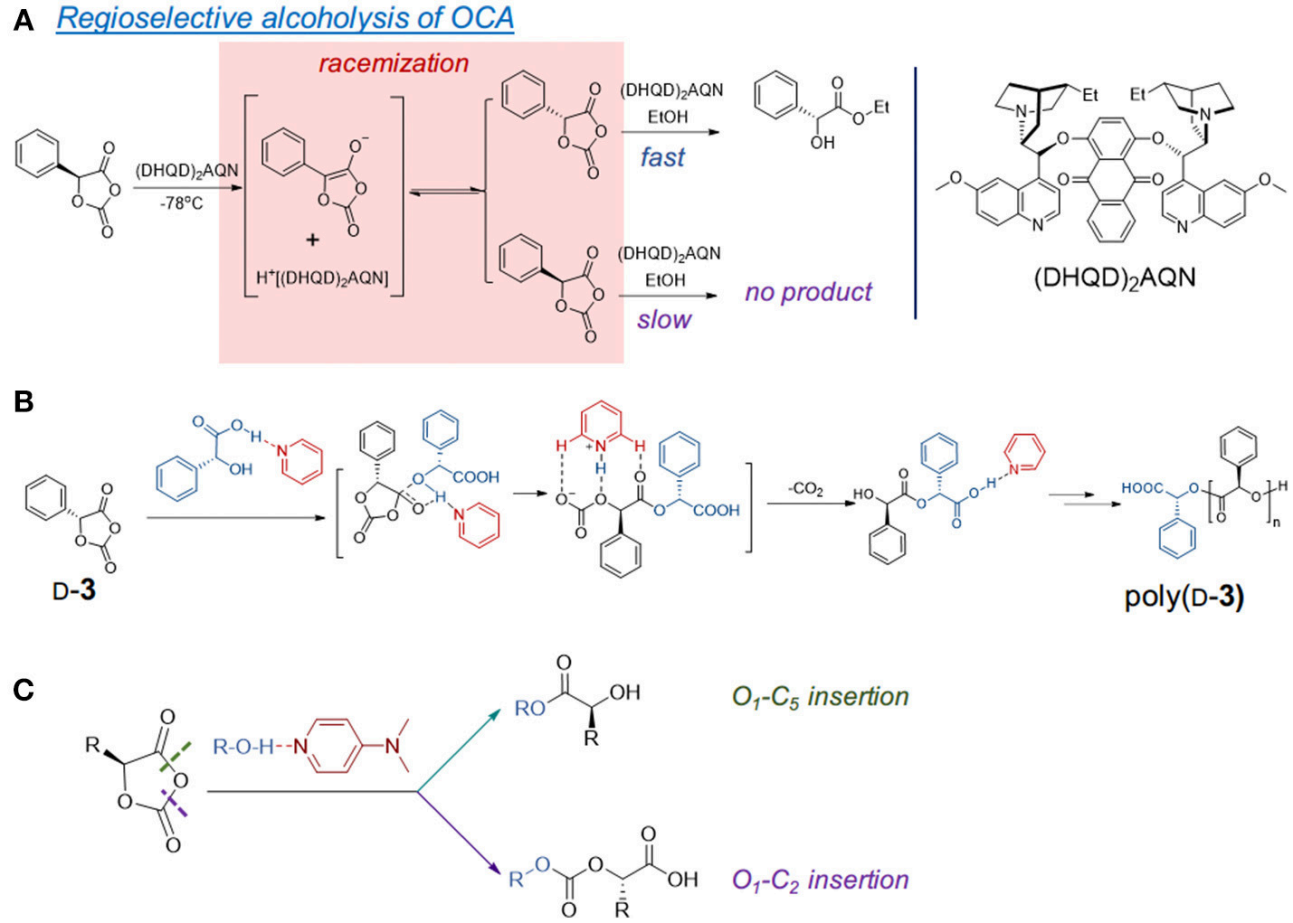
Epimerization of α-proton in the organocatalyst-mediated OCA polymerization. **(A)** The reported racemization of aryl-OCA (**3**) at low temperature in the presence of amine. **(B)** The use of D-mandelic acid/pyridine crystalline adduct can suppress the epimerization and provide isotactic-dominant poly(D-**3**). **(C)** Another proposed mechanism of DMAP/alcohol induced epimerization by non-regioselective ring-opening of OCA.

Early studies by Kricheldorf and Jonté showed that the ROP of l-**1** mediated by bases was accompanied by epimerization, as the optical rotations of the polymers decreased with the increase of the catalyst basicity (Kricheldorf and Jont, 1983). The racemization of α-proton in the 5-aryl-OCA monomers (e.g., **3**) was also found in the alcoholysis mediated by a modified cinchona alkaloid, an aprotic nucleophile bearing tertiary amine and quinoline (Figure 2A) at −70°C (Tang and Deng, 2002). The kinetic studies showed that the interconversion between *S*- and *R*-**3** was much faster than the enantioselective alcoholysis. When the aryl groups were replaced by alkyl groups, the reduced acidity of the α-proton rendered it unepimerizable by the cinchona alkaloid catalyst, which suggested the importance of the electronic property of the functional group on the 5-position of OCA monomers (Tang and Deng, 2002).

However, recent studies showed that epimerization of the α-proton still occurred to the DMAP-mediated ROP of OCAs bearing alkyl groups (e.g., **4** and **5**) (Pounder et al., 2011; Wang et al., 2016). The decreased epimerization of the α-proton occurred in poly(l-**4**) when DMAP (p*K*_a_ = 9.7) was replaced by less basic 4-methoxypyridine (p*K*_a_ = 6.6) (Pounder et al., 2011). Similarly, the improved isotacticity of the poly(d-**3)** was found by using pyridine derivatives with decreased basicity (Buchard et al., 2014).

The use of acid/base crystalline adducts of mandelic acid and pyridine for the ROP of d-**3** could suppress the racemization, and thereby (Figure 2B) could produce highly stereoregular isotactic polymers up to 48.0 kDa (over 80 h with Ð^*^ of 1.17), which display enhanced thermal properties compared with the atactic poly(**3**) (Buchard et al., 2014). Similarly, the adducts of 4-methoxypyridine with l-lactic acid and β-benzyl α-l-malate could also initiate ROP of l-**1** minimized epimerization of α-protons; however, those adducts failed to provide isotactic poly(l-**4**) (Bexis et al., 2017). Note that at low [OCA]/[initiator] ratios, epimerization still occurred in both polymers when using the acid-base adducts, suggesting that the pyridine adduct can still lead to epimerization even with decreased basicity (Bexis et al., 2017).

### Lewis Pair Catalyst for OCA Polymerization

The use of a Lewis pair complex, that is a combination of Lewis acid with a base, has achieved recent success in controlled linear polymerization of acrylate, lactones and the synthesis of cyclic poly(lactide) (Hong et al., 2018). A very recent report showed that the use of the Lewis pair of Zn(C_6_F_5_)_2_ with primary or secondary amines could initiate the polymerization of l-**2** and l-**3** (Nie et al., 2018). The obtained polymers had *M*_n_s up to 26.8 kDa with *-D*s < 1.1. However, severe epimerization (isotacticity <80%) occurred in both polymers (Nie et al., 2018). The use of bases in the Lewis pairs could therefore be detrimental to obtaining stereo-regular polymers from OCAs. Note that the same Lewis pair afforded cyclic PLAs, presumably via the zwitterionic intermediate with Zn(C_6_F_5_)_2_ and amine on each polymer chain terminus (Piedra-Arroni et al., 2013). The discrepancy between the two ROPs by the same Lewis pair indicated that chain propagation in the polymerization of OCAs was dominantly mediated by the Zn moiety without the influence of the amine. This was also attributed to the relatively low MWs, similar to those polymerizations promoted only by Zn-alkoxides (Wang et al., 2016; Feng and Tong, 2017a), which is discussed section Metal catalyst for OCA polymerization.

### Other Concerns in Organocatalyst-Mediated OCA Polymerization

Computational studies by Bourissou et al. proposed that DMAP acts in a bifunctional nature by activating both the initiating alcohol and the carboxy oxygen O_5_ in OCA (Bonduelle et al., 2008). However, another computational study hinted that the pyridine-catalyzed ROP of OCAs could occur in both O_1_-C_5_ (ester formation) and O_1_-C_2_ (carbonate formation), which probably leads to epimerization (Figure 2C). Besides these computation studies, no detailed mechanistic studies revealed the initiation and chain propagation for DMAP or pyridine-analog mediated ROP. There also lacks the kinetic studies to reflect the reactivity order of DMAP and *k*_app_ during chain propagation.

In addition, studies using most organocatalysts to promote the ROP of OCAs usually started from **1**, assuming that the success in **1** can be translated to other OCAs meaningfully. As observed in the ROP of lactones, when the methyl group of lactide is replaced with other groups, the polymerization conditions became harsh with incomplete monomer conversions and low DPs (Pounder and Dove, 2010; Chen et al., 2014). The ROP of OCAs using organocatalysts is similar. We found that at a high monomer-to-initiator ratio (500), the combination of DMAP/BnOH was not able to efficiently initiate the polymerization of l-**2** (conversion of **2** = 57% in 24 h), in contrast to the results of the ROP of l-**1** using the same catalysts (Feng and Tong, 2017a). Similarly, incomplete conversion of l-**2** with a low MW (90% in 24 h, *M*_n_ = 3.2 kDa, *-D* = 2.19) was found in the reaction catalyzed by NHC/BnOH (NHC, 1,3-bis(2,6-diisopropylphenyl)imidazol-2-ylidene) (Feng and Tong, 2017a). As the motivation of studying OCA polymerization is to synthesize polyesters with pendant functional groups, we suggest that researchers should start from the OCA monomers bearing functional groups (e.g., **2**) and validate the results in other monomers, instead of only reporting the results from **1**.

## Metal Catalyst for OCA Polymerization

### Development of Metal Catalysts

**Figure 3:**
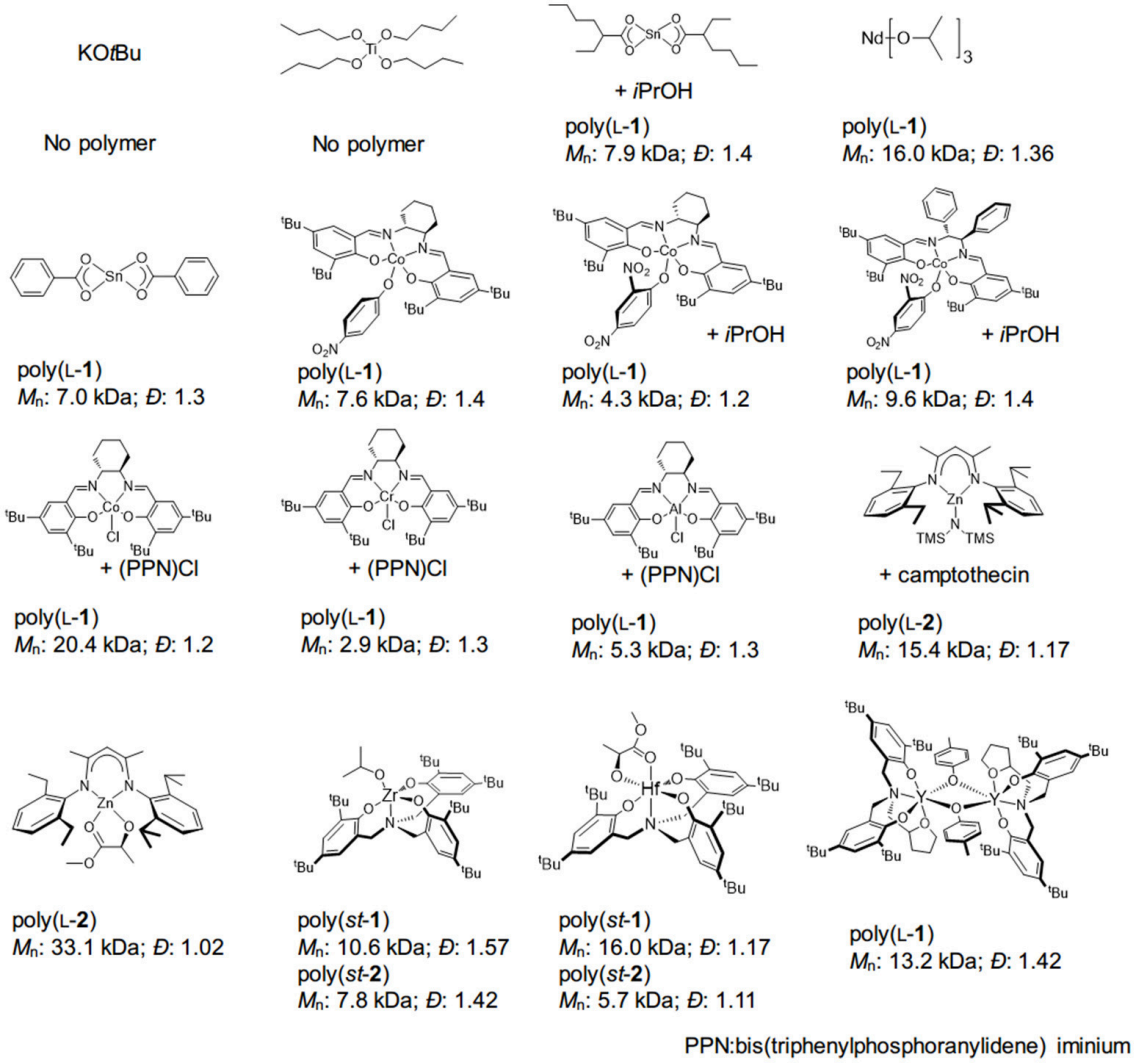
Representative results of various metal catalysts-mediated OCA polymerization. st, syndiotactic.

Compared with the organocatalysts, the development of organometallic catalysts in OCA polymerization is surprisingly slow. Many organometallics that successfully mediated the ROP of lactide, lactones, and NCAs failed to translate to the polymerization of OCAs. Metal complexes that can promote ROPs of lactones or the copolymerization of epoxides and CO_2_, including Ti(IV), K, Sn(II), Al(III), Co(III), Nd(III), and Cr(III) complexes, did not mediate controlled ROP of l-**1** (Figure 3; Kricheldorf and Jont, 1983; Zhuang et al., 2010; He et al., 2013; Jia et al., 2015).

The Zn complexes with β-diiminate (BDI) ligands represented the first metal catalyst system that could mediate controlled ROP of OCAs (Figure 3; Yin et al., 2013; Wang et al., 2016). Similar to the well-known ROP of lactide (Chamberlain et al., 2001), the BDI-Zn complex requires an alcohol, even a very bulky one such as camptothecin or PEG (Yin et al., 2013), to promote polymerization, presumably through a coordination-insertion mechanism (Wang et al., 2016). It is worth noting that the monomeric BDI-Zn/alcohol complex performs more efficiently than a dimeric complex, which also agrees well with the reaction rate obtained in kinetic studies (Wang et al., 2016). In addition, no epimerization was found in the ROP of OCAs (for **2, 3, 5**), indicating that no nucleophilic attack toward α-protons occurred for BDI-Zn complexes (Wang et al., 2016). As BDI-Zn-alkoxide can mediate the polymerization of either lactone or OCA, the sequential polymerization of lactone (including lactide) with OCAs can be smoothly achieved, regardless of monomer addition sequence (Wang et al., 2016).

Nevertheless, the BDI-Zn/alcohol complex cannot efficiently produce polymers with a high DP (≥300) (Feng and Tong, 2017a). This may be due to inefficient chain propagation; the insertion of Zn-alkoxide into l-**2** was not followed by immediate decarboxylation, resulting in the inactive Zn-carbonate species at the chain end (Feng and Tong, 2017a). The mechanistic studies of using BDI-Zn to copolymerize epoxide and CO_2_ indicate the equilibrium between Zn-alkoxide and Zn-carbonate (Cheng et al., 2001; Moore et al., 2003; Jeske et al., 2007; Longo et al., 2016). Note that very recent studies involving the use of Zr, Hf (Sun et al., 2017), La and Y (Ouyang et al., 2017) complexes did not intend for high-MW polyester synthesis (Figure 3). For the reasons alluded to above (section Molecular weight of the polyester on the polymer MW), it is therefore crucial to develop a highly efficient decarboxylation process for rapid chain propagation in OCA polymerization.

**Figure 4:**
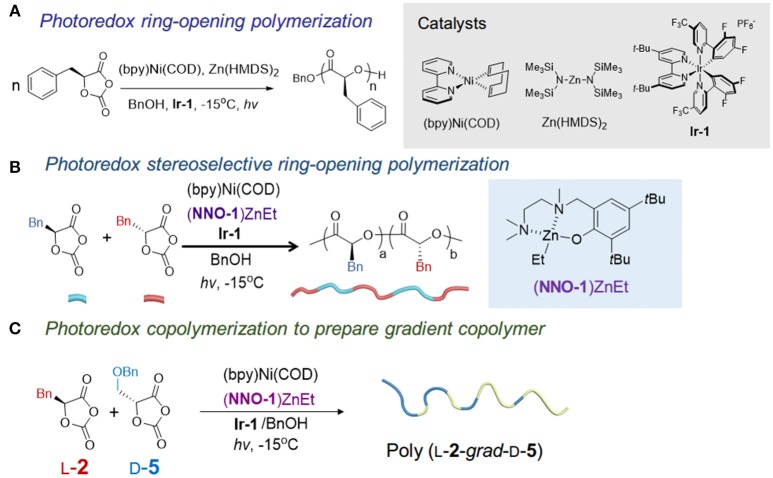
Photoredox polymerizations of OCAs. **(A)** The photoredox ROP of OCAs to prepare high MW isotactic polymers using (bpy)Ni/Zn(HMDS)2/Ir-1 catalysts. **(B)** Photoredox stereoselective copolymerization of racemic OCAs to prepare stereoblock polymers. **(C)** Photoredox copolymerization of two monomers with opposite chirality and significantly different reactivities to synthesize gradient copolymer. *hv*, blue LED with wavelengths of 400–500 nm.

We noticed that many metal catalysts for lactone polymerization, such as BDI-Zn, are disqualified for decarboxylation as they have been also used for polycarbonate synthesis (e.g., Al, Fe, Cr, Co) (Lu X. B. et al., 2012; Paul et al., 2015; Tong, 2017) (as have many organocatalysts Kiesewetter et al., 2010). Aware of substantial studies on metal catalyst-mediated NCA polymerization (Deming, 1997, 1998; Deming and Curtin, 2000) and the recent surge of interest in the photoredox catalysis (Prier et al., 2013; Zuo et al., 2014; Le and MacMillan, 2015), we developed a protocol for controlled photoredox ROP of enantiopure OCAs (**1, 2, 5, 6**) to afford isotactic polyesters with high MWs (>140 kDa) and narrow *- D*s (<1.1) without epimerization at the α-methine hydrogen (Figure 4A) (Feng and Tong, 2017a). In such a system, the combination of the (bpy)Ni(0) complex (bpy, 2,2'-bipyridyl), a catalyst for NCA polymerization (Deming, 1997, 1998) but not reactive for controlled OCA polymerization *per se* (Feng and Tong, 2017a), and the photoredox catalyst **Ir-1**, is employed to efficiently promote the decarboxylation process under light irradiation based on the decarboxylation mechanism reported by the MacMillan lab (Zuo et al., 2014; Le and MacMillan, 2015). Zn(HMDS)_2_ was identified after screening a number of Zn complexes whereas the bulky BDI-Zn complexes do not provide high-MW polymers in the photoredox setting (Feng and Tong, 2017a). Kinetic studies indicated that the use of alcohol was only involved in the initiation to form Zn-alkoxide for ring-opening reactions and **Ir-1** only influenced the Ni complex's oxidative state but did not affect chain-end reactivity. Mechanistic studies suggested that a Ni(0) complex regioselectively inserted at the O_1_-C_5_ bond in the OCA monomer, followed by Ir-mediated photoredox decarboxylation and transmetalation with a Zn complex, formed a reactive Zn-alkoxide terminus for chain propagation (Feng and Tong, 2017a). Notably, the polymerization has to be performed at low temperature (−15 to 20°C) to avoid the undesired Ni-mediated decarbonylation that occurs at room temperature (Yamamoto et al., 1980; Sano et al., 1984; Johnson et al., 2007).

### Stereoselective ROP by Metal Catalysts

Organometallic catalysts are advantageous for preparing polyesters with various microstructures from lactides and β-lactones (Ajellal et al., 2010; Carpentier, 2010; Stanford and Dove, 2010; Thomas, 2010), although there have been reports on the utilization of organocatalysts (Dove et al., 2006; Zhang et al., 2007; Zhu and Chen, 2015). Nevertheless, the stereoselective synthesis of polyesters with pendant side-chain functional groups remains challenging. In 2017, Wu et al. identified a Hf-alkoxide complex with a *C*_3_ symmetric amino-tris(phenolate) ligand for syndioselective ROP of racemic OCAs (**1, 2**, and **7**, Figure 3) (Sun et al., 2017). Most obtained polymers have MWs <20 kDa with a relatively broad *- D* (>1.1). The origin of such syndioselectivity (chirality influence of the metal), and the chain propagation mechanism, was not well-understood [chain-end or enantiomorphic mechanism; see the discussion on the Ge complex with the same *C*_3_ symmetric for lactide polymerization (Chmura et al., 2007, 2008)]. The Hf complex was utilized to mediate alternative copolymerization of the co-monomers with opposite chirality; however, all obtained copolymers had relatively low MWs (<10 kDa), and monomer conversions were even incomplete in many cases (Sun et al., 2017).

We have recently applied the photoredox Ni/Zn catalysts to stereoselective polymerization of OCAs (Feng et al., 2018). A (**NNO-1**)Zn complex with less bulky tridentate Schiff base ligands, compared with BDI, was identified to mediate the stereoselective and controlled polymerization of racemic OCAs (**1**, **2**, **5**, **6**) that afforded stereoblock polymers (Figure 4B). The obtained stereoblock copolymers are highly isotactic with high MWs (>70 kDa) and narrow Ð^*^s (*M*_w_/*M*_n_ < 1.1), with a probability of *meso* dyad formation (*P*_m_, i.e., isotactic enchainment) of 0.97. To elucidate the microstructures and polymerization mechanism, deuterated [D_2_]-l-**2** was synthesized from [D_2_]-l-phenylalanine and used for kinetic studies together with d-**2** in the polymerization (Feng et al., 2018), as the methine deuterium in [D_2_]-l-**2** does not show up in the ^1^H NMR spectrum. The NMR results suggested the polymer chain end did not have a kinetic preference for a specific enantiomer in the (**NNO-1**)ZnEt-mediated photoredox ROP of *rac*-**2**. Different from the enantiomorphic site-control by using racemic chiral aluminum catalysts for stereoblock PLA synthesis, the (bpy)Ni/(**NNO-1**)Zn/**Ir-1** mediated stereoselective ROPs proceeds via the chain-end control; a stereoerror occurs during the chain propagation and the other enantiomer is incorporated and enchained (Feng et al., 2018). The computational study suggests the stereo-hindrance in the **NNO-1** ligand affected the isoselectivity of the Zn complex. Notably, the obtained stereoblock polymers exhibited melting temperatures close to the stereocomplex of two isotactic polymers (Feng et al., 2018).

Subsequent copolymerization studies expand the use of (**NNO-1**) Zn photoredox system for the gradient copolymer synthesis. We found that using monomers with opposite chirality and significant difference in the polymerization rates (i.e., *k*_app_) result in the formation of gradient copolymers with *M*_n_s close to the calculated MWs (over 40 kDa) and Ð^*^ values of < 1.1 (Figure 4C). The polymerization rates for OCA monomers in Ni/Zn systems have the following orders: *k*(**2**) **>** *k*(**1**) ≈ *k*(**6**) **>** *k*(**5**). No obvious difference exists between the rates of the two enantiomers of a given monomer. On the other hand, copolymerizations of monomers with the same chirality or similar polymerization rates can lead to the random copolymers.

## Perspective

Despite the substantial number of catalysts that have been developed (Feng et al., 2017), few have really held promise for industrial production or commercialization. It remains difficult for organocatalysts to mediate stereoregular polymerization from OCAs as the racemization of α-proton persists when using most bases, even at low temperature (Tang and Deng, 2002) or with decreased basicity (Bexis et al., 2017). The detailed chain-propagation mechanism, including non-regioselective ring-opening of OCA (Pounder et al., 2011) and the existence of an active-monomer mechanism (Kricheldorf and Jont, 1983; Bonduelle et al., 2008), has not been well-studied compared with those results in the ROP of NCAs (Kricheldorf, 2006; Hadjichristidis et al., 2009; Cheng and Deming, 2012). On the other hand, substantial progress has been achieved in the use of metal complexes for controlled OCA polymerization, which allows for the synthesis of high MW polymers and stereoselective polymerizations (Feng and Tong, 2017a; Feng et al., 2018). However, the use of low temperature and relatively exotic experimental conditions could prevent the direct translation of the photoredox Ni/Zn catalysts to industrial production. Our review is not able to impart a perfect ability to predict what will work: the lessons learned in one context (e.g., polymerizations of lactide or NCAs) do not always translate into the ROP of OCAs, even when one might expect them to. Such instances reflect the fact that our understanding of the OCA polymerization mechanism remains incomplete.

Notably, as has been the case for decades, a comparison of results from different catalytic systems remains difficult, with many inconclusive or incomprehensive studies. The standardized and systemic studies can be helpful for future chemistry development, together with the use of visualized experimental procedures (Feng and Tong, 2017b).

Additionally, as many new polymers have been synthesized from OCAs, it is important to characterize their physiochemical properties, including their degradation profiles, to identify their potential applications. It is also important to start to design and perform studies on how to recycle the polymers for sustainable applications (Hillmyer and Tolman, 2014; Hong and Chen, 2017; Rahimi and García, 2017; Schneiderman and Hillmyer, 2017; Sardon and Dove, 2018; Zhu et al., 2018). Irrespective of the industrial prospects for the polyesters, the rapidly developing OCA chemistry can be suggestive for other polymerizations, in such a way as it benefits from the great expansion of the polymer field.

## Author Contributions

All authors listed have made a substantial, direct and intellectual contribution to the work, and approved it for publication.

### Conflict of Interest Statement

The authors declare that the research was conducted in the absence of any commercial or financial relationships that could be construed as a potential conflict of interest.

## References

[B1] Ajellal N. Carpentier J.-F. Guillaume C. Guillaume S. M. Helou M. Poirier V.. (2010). Metal-catalyzed immortal ring-opening polymerization of lactones, lactides and cyclic carbonates. Dalton Trans. 39, 8363–8376. 10.1039/c001226b 20424735

[B2] Anderson K. S. Schreck K. M. Hillmyer M. A. (2008). Toughening polylactide. Polym Rev. 48, 85–108. 10.1080/15583720701834216

[B3] Armentano I. Bitinis N. Fortunati E. Mattioli S. Rescignano N. Verdejo R. (2013). Multifunctional nanostructured PLA materials for packaging and tissue engineering. Prog. Polym. Sci. 38, 1720–1747. 10.1016/j.progpolymsci.2013.05.010

[B4] Auras R. Harte B. Selke S. (2004). An overview of polylactides as packaging materials. Macromol. Biosci. 4, 835–864. 10.1002/mabi.200400043 15468294

[B5] Becker G. Wurm F. R. (2018). Functional biodegradable polymers via ring-opening polymerization of monomers without protective groups. Chem. Soc. Rev. 47, 7739–7782. 10.1039/C8CS00531A 30221267

[B6] Bexis P. De Winter J. Coulembier O. Dove A. P. (2017). Isotactic degradable polyesters derived from O-carboxyanhydrides of l-lactic and l-malic acid using a single organocatalyst/initiator system. Eur. Polym. J. 95, 660–670. 10.1016/j.eurpolymj.2017.05.038

[B7] Bonduelle C. Martín-Vaca B. Cossío F. P. Bourissou D. (2008). Monomer versus alcohol activation in the 4-Dimethylaminopyridine-Catalyzed Ring-opening polymerization of lactide and Lactic O-Carboxylic Anhydride. Chem. Eur. J. 14, 5304–5312. 10.1002/chem.200800346 18446916

[B8] Bourissou D. Moebs-Sanchez S. Martín-Vaca B. (2007). Recent advances in the controlled preparation of poly(α-hydroxy acids): metal-free catalysts and new monomers. Comptes Rendus Chim. 10, 775–794. 10.1016/j.crci.2007.05.004

[B9] Breslow D. S. Hulse G. E. Matlack A. S. (1957). Synthesis of Poly-β-alanine from Acrylamide. A novel synthesis of β-Alanine1. J. Am. Chem. Soc. 79, 3760–3763. 10.1021/ja01571a039

[B10] Buchard A. Carbery D. R. Davidson M. G. Ivanova P. K. Jeffery B. J. Kociok-Köhn G. I.. (2014). Preparation of Stereoregular Isotactic Poly(mandelic acid) through Organocatalytic ring-opening polymerization of a cyclic O-carboxyanhydride. Angew. Chem. Int. Chem. 53, 13858–13861. 10.1002/anie.201407525 25314676

[B11] Carpentier J.-F. (2010). Discrete metal catalysts for stereoselective ring-opening polymerization of chiral Racemic β-Lactones. Macromol. Rapid Commun. 31, 1696–1705. 10.1002/marc.201000114 21567583

[B12] Chamberlain B. M. Cheng M. Moore D. R. Ovitt T. M. Lobkovsky E. B. Coates G. W. (2001). Polymerization of lactide with zinc and magnesium β-Diiminate complexes: stereocontrol and mechanism. J. Am. Chem. Soc. 123, 3229–3238. 10.1021/ja003851f 11457057

[B13] Chen X. Lai H. Xiao C. Tian H. Chen X. Tao Y. (2014). New bio-renewable polyester with rich side amino groups from l-lysine via controlled ring-opening polymerization. Polym. Chem. 5, 6495–6502. 10.1039/C4PY00930D

[B14] Cheng J. Deming T. J. (2012). Synthesis of polypeptides by ring-opening polymerization of α-Amino Acid N-Carboxyanhydrides, in Peptide-Based Materials, ed Deming T. (Berlin; Heidelberg: Springer), 1–26. 10.1007/128_2011_173 21647839

[B15] Cheng M. Moore D. R. Reczek J. J. Chamberlain B. M. Lobkovsky E. B. Coates G. W. (2001). Single-site β-Diiminate zinc catalysts for the alternating copolymerization of CO2 and epoxides: catalyst synthesis and unprecedented polymerization activity. J. Am. Chem. Soc. 123, 8738–8749. 10.1021/ja003850n 11535078

[B16] Chmura A. J. Chuck C. J. Davidson M. G. Jones M. D. Lunn M. D. Bull S. D.. (2007). A germanium alkoxide supported by a C3-symmetric ligand for the stereoselective synthesis of highly heterotactic polylactide under solvent-free conditions. Angew. Chem. Int. Ed. 46, 2280–2283. 10.1002/anie.200603944 17299819

[B17] Chmura A. J. Davidson M. G. Frankis C. J. Jones M. D. Lunn M. D. (2008). Highly active and stereoselective zirconium and hafnium alkoxide initiators for solvent-free ring-opening polymerization of rac-lactide. Chem. Commun. 1293–1295. 10.1039/b718678a 18389111

[B18] Danhier F. Ansorena E. Silva J. M. Coco R. Le Breton A. Préat V. (2012). PLGA-based nanoparticles: an overview of biomedical applications. J. Control. Release 161, 505–522. 10.1016/j.jconrel.2012.01.043 22353619

[B19] Davies W. H. (1951). 302. Anhydrocarboxy-derivatives of hydroxy- and mercapto-acids. J. Chem. Soc 1357–1359.

[B20] Dechy-Cabaret O. Martin-Vaca B. Bourissou D. (2004). Controlled ring-opening polymerization of lactide and glycolide. Chem. Rev. 104, 6147–6176. 10.1021/cr040002s 15584698

[B21] Degée P. Dubois P. Jérome R. Jacobsen S. Fritz H.-G. (1999). New catalysis for fast bulk ring-opening polymerization of lactide monomers. Macromol. Symp. 144, 289–302. 10.1002/masy.19991440126

[B22] Deming T. J. (1997). Facile synthesis of block copolypeptides of defined architecture. Nature 390, 386–389. 9389476 10.1038/37084

[B23] Deming T. J. (1998). Amino acid derived nickelacycles: intermediates in nickel-mediated polypeptide synthesis. J. Am. Chem. Soc. 120, 4240–4241. 10.1021/ja980313i

[B24] Deming T. J. Curtin S. A. (2000). Chain initiation efficiency in cobalt- and nickel-mediated polypeptide synthesis. J. Am. Chem. Soc. 122, 5710–5717. 10.1021/ja994281q

[B25] Di Y. W. Iannace S. Di Maio E. Nicolais L. (2005). Poly(lactic acid)/organoclay nanocomposites: thermal, rheological properties and foam processing. J. Polym. Sci. B 43, 689–698. 10.1002/polb.20366

[B26] Dove A. P. Li H. B. Pratt R. C. Lohmeijer B. G. G. Culkin D. A. Waymouth R. M.. (2006). Stereoselective polymerization of rac- and meso-lactide catalyzed by sterically encumbered N-heterocyclic carbenes. Chem. Commun. 2881–2883. 10.1039/b601393g 17007404

[B27] du Boullay O. T. Bonduelle C. Martin-Vaca B. Bourissou D. (2008). Functionalized polyesters from organocatalyzed ROP of gluOCA, the O-carboxyanhydride derived from glutamic acid. Chem. Commun. 1786–1788. 10.1039/b800852c 18379693

[B28] du Boullay O. T. Marchal E. Martin-Vaca B. Cossio F. P. Bourissou D. (2006). An activated equivalent of lactide toward organocatalytic ring-opening polymerization. J. Am. Chem. Soc. 128, 16442–16443. 10.1021/ja067046y 17177360

[B29] Dubois P. Jacobs C. Jerome R. Teyssie P. (1991). Macromolecular engineering of polylactones and polylactides. 4. Mechanism and kinetics of lactide homopolymerization by aluminum isopropoxide. Macromolecules 24, 2266–2270. 10.1021/ma00009a022

[B30] Duda A. Kowalski A. Libiszowski J. Penczek S. (2005). Thermodynamic and kinetic polymerizability of cyclic esters. Macromol. Symp. 224, 71–84. 10.1002/masy.200550607

[B31] Farah S. Anderson D. G. Langer R. (2016). Physical and mechanical properties of PLA, and their functions in widespread applications — A comprehensive review. Adv. Drug Deliv. Rev. 107, 367–392. 10.1016/j.addr.2016.06.012 27356150

[B32] Feng Q. Tong R. (2017a). Controlled photoredox ring-opening polymerization of *O*-Carboxyanhydrides. J. Am. Chem. Soc. 139, 6177–6182. 10.1021/jacs.7b01462 28397499

[B33] Feng Q. Tong R. (2017b). Controlled photoredox ring-opening polymerization of O-carboxyanhydrides mediated by Ni/Zn complexes. J. Vis. Exp. e56654. 10.3791/56654 29286388 PMC5755454

[B34] Feng Q. Yang L. Zhong Y. Guo D. Liu G. Xie L.. (2018). Stereoselective photoredox ring-opening polymerization of O-carboxyanhydrides. Nat. Commun. 9:1559. 10.1038/s41467-018-03879-5 29674720 PMC5908805

[B35] Feng Q. Zhong Y. Xie L. Tong R. (2017). Recent advances in ring-opening polymerization of O-Carboxyanhydrides. Synlett 28, 1857–1866. 10.1055/s-0036-1590841

[B36] Garlotta D. A. (2001). Literature review of Poly(Lactic Acid). J. Polym. Environ. 9, 63–84. 10.1023/A:1020200822435

[B37] Gregory G. L. López-Vidal E. M. Buchard A. (2017). Polymers from sugars: cyclic monomer synthesis, ring-opening polymerisation, material properties and applications. Chem. Commun. 53, 2198–2217. 10.1039/C6CC09578J 28127607

[B38] Grubbs R. B. Grubbs R. H. (2017). 50th anniversary perspective: living polymerization—emphasizing the molecule in macromolecules. Macromolecules 50, 6979–6997. 10.1021/acs.macromol.7b01440

[B39] Hadjichristidis N. Iatrou H. Pitsikalis M. Sakellariou G. (2009). Synthesis of well-defined polypeptide-based materials via the ring-opening polymerization of α-Amino Acid N-Carboxyanhydrides. Chem. Rev. 109, 5528–5578. 10.1021/cr900049t 19691359

[B40] He Z. Jiang L. Chuan Y. Li H. Yuan M. (2013). Ring-opening polymerization of l-Lactic Acid O-Carboxyanhydrides initiated by alkoxy rare earth compounds. Molecules 18, 12768–12776. 10.3390/molecules181012768 24132199 PMC6270375

[B41] Hillmyer M. A. Tolman W. B. (2014). Aliphatic polyester block polymers: renewable, degradable, and sustainable. Acc. Chem. Res. 47, 2390–2396. 10.1021/ar500121d 24852135

[B42] Hong M. Chen E. Y. X. (2017). Chemically recyclable polymers: a circular economy approach to sustainability. Green Chem. 19, 3692–3706. 10.1039/C7GC01496A

[B43] Hong M. Chen J. Chen E. Y. X. (2018). Polymerization of polar monomers mediated by main-group lewis Acid–base pairs. Chem. Rev. 118, 10551–10616 10.1021/acs.chemrev.8b00352 30350583

[B44] Houk K. N. Jabbari A. Hall H. K. Alemán C. (2008). Why δ-valerolactone polymerizes and γ-Butyrolactone does not. J. Org. Chem. 73, 2674–2678. 10.1021/jo702567v 18324833

[B45] Jacobsen S. Fritz H. G. Degée P. Dubois P. Jérôme R. (1999). Polylactide (PLA)—a new way of production. Polym. Eng. Sci. 39, 1311–1319. 10.1002/pen.11518

[B46] Jambeck J. R. Geyer R. Wilcox C. Siegler T. R. Perryman M. Andrady A.. (2015). Plastic waste inputs from land into the ocean. Science, 347, 768–771. 10.1126/science.1260352 25678662

[B47] Jeske R. C. DiCiccio A. M. Coates G. W. (2007). Alternating copolymerization of epoxides and cyclic anhydrides: an improved route to aliphatic polyesters. J. Am. Chem. Soc. 129, 11330–11331. 10.1021/ja0737568 17722928

[B48] Jia F. Chen X. Zheng Y. Qin Y. Tao Y. Wang X. (2015). One-pot atom-efficient synthesis of bio-renewable polyesters and cyclic carbonates through tandem catalysis. Chem. Commun. 51, 8504–8507. 10.1039/C5CC01329A 25892206

[B49] Johnson J. B. Bercot E. A. Rowley J. M. Coates G. W. Rovis T. (2007). Ligand-dependent catalytic cycle and role of styrene in nickel-catalyzed anhydride cross-coupling: evidence for turnover-limiting reductive elimination. J. Am. Chem. Soc. 129, 2718–2725. 10.1021/ja067845g 17295486

[B50] Kiesewetter M. K. Shin E. J. Hedrick J. L. Waymouth R. M. (2010). Organocatalysis: opportunities and challenges for polymer synthesis. Macromolecules 43, 2093–2107. 10.1021/ma9025948

[B51] Kowalski A. Duda A. Penczek S. (2000a). Mechanism of cyclic ester polymerization initiated with Tin(II) Octoate. 2. Macromolecules fitted with Tin(II) Alkoxide species observed directly in MALDI–TOF spectra. Macromolecules 33, 689–695. 10.1021/ma9906940

[B52] Kowalski A. Libiszowski J. Duda A. Penczek S. (2000b). Polymerization of l,l-Dilactide Initiated by Tin(II) Butoxide. Macromolecules 33, 1964–1971. 10.1021/ma991751s

[B53] Kramer J. R. Deming T. J. (2010). General method for purification of α-Amino acid-N-carboxyanhydrides using flash chromatography. Biomacromolecules 11, 3668–3672. 10.1021/bm101123k 21047056

[B54] Kricheldorf H. R. (2006). Polypeptides and 100 years of chemistry of α-Amino Acid N-Carboxyanhydrides. Angew. Chem. Int. Chem. 45, 5752–5784. 10.1002/anie.200600693 16948174

[B55] Kricheldorf H. R. Jonté J. M. (1983). New polymer syntheses. 8. Synthesis and polymerization of l-Lactic Acid O-Carboxyanhydride (5-Methyl-Dioxolan-2,4-dione). Polym. Bull. 9, 276–283. 10.1007/BF00262719

[B56] Kühnert I. Spörer Y. Brünig H. Tran N. H. A. Rudolph N. (2018). Processing of Poly(lactic Acid), in Industrial Applications of Poly(lactic acid), eds Di Lorenzo M. L. Androsch R. (Cham: Springer International Publishing), 1–33.

[B57] Lasprilla A. J. R. Martinez G. A. R. Lunelli B. H. Jardini A. L. Filho R. M. (2012). Poly-lactic acid synthesis for application in biomedical devices — A review. Biotechnol. Adv. 30, 321–328. 10.1016/j.biotechadv.2011.06.019 21756992

[B58] Le C. MacMillan D. W. C. (2015). Fragment couplings via CO2 extrusion-recombination: expansion of a classic bond-forming strategy via metallaphotoredox. J. Am. Chem. Soc. 137, 11938–11941. 10.1021/jacs.5b08304 26333771 PMC4632494

[B59] Lim L. T. Auras R. Rubino M. (2008). Processing technologies for poly(lactic acid). Prog. Polym. Sci. 33, 820–852. 10.1016/j.progpolymsci.2008.05.004

[B60] Longo J. M. Sanford M. J. Coates G. W. (2016). Ring-opening copolymerization of epoxides and cyclic anhydrides with discrete metal complexes: structure–property relationships. Chem. Rev. 116, 15167–15197. 10.1021/acs.chemrev.6b00553 27936619

[B61] Lu X. B. Ren W. M. Wu G. P. (2012). CO2 copolymers from epoxides: catalyst activity, product selectivity, and stereochemistry control. Acc. Chem. Res. 45, 1721–1735. 10.1021/ar300035z 22857013

[B62] Lu Y. Yin L. Zhang Y. Zhang Z. Xu Y. Tong R.. (2012). Synthesis of Water-Soluble Poly(α-hydroxy acids) from Living Ring-opening polymerization of O-Benzyl-l-serine carboxyanhydrides. ACS Macro Lett. 1, 441–444. 10.1021/mz200165c 23359651 PMC3555137

[B63] Martin Vaca B. Bourissou D. (2015). O-Carboxyanhydrides: useful tools for the preparation of well-defined functionalized polyesters. ACS Macro Lett. 4, 792–798. 10.1021/acsmacrolett.5b00376 35596481

[B64] Middleton J. C. Tipton A. J. (2000). Synthetic biodegradable polymers as orthopedic devices. Biomaterials 21, 2335–2346. 10.1016/S0142-9612(00)00101-0 11055281

[B65] Moore D. R. Cheng M. Lobkovsky E. B. Coates G. W. (2003). Mechanism of the alternating copolymerization of epoxides and CO2 using β-Diiminate zinc catalysts: evidence for a bimetallic epoxide enchainment. J. Am. Chem. Soc. 125, 11911–11924. 10.1021/ja030085e 14505413

[B66] Nederberg F. Connor E. F. Möller M. Glauser T. Hedrick J. L. (2001). New paradigms for organic catalysts: the first organocatalytic living polymerization. Angew. Chem. Int. Chem. 40, 2712–2715. 10.1002/1521-3773(20010716)40:14<2712::AID-ANIE2712>3.0.CO;2-Z 29712329

[B67] Nie Y. Wang P. Du H. Meng W. Yang J. (2018). An efficient strategy for achieving controlled ring-opening polymerization of O-carboxyanhydrides via amine initiation in collaboration with metal-alkoxide catalysis. Polym. Chem. 9, 5014–5023. 10.1039/C8PY01090K

[B68] Nofar M. Park C. B. (2014). Poly (lactic acid) foaming. Prog. Polym. Sci. 39, 1721–1741. 10.1016/j.progpolymsci.2014.04.001

[B69] Odian G. (2004). Ring-opening polymerization, in Principle of Polymerization (Hoboken, NJ: John Wiley & Sons, Inc.), 544–618.

[B70] Otake Y. Nakamura H. Fuse S. (2018). Rapid and mild synthesis of amino acid n-carboxy anhydrides: basic-to-acidic flash switching in a microflow reactor. Angew. Chem. Int. Chem. 57, 11389–11393. 10.1002/anie.201803549 29998576

[B71] Ouyang H. Nie K. Yuan D. Yao Y. (2017). Synthesis of amine-bridged bis(phenolate) rare-earth metal aryloxides and their catalytic performances for the ring-opening polymerization of l-lactic acid O-carboxyanhydride and l-lactide. Dalton Trans. 46, 15928–15938. 10.1039/C7DT03001K 29119172

[B72] Paul S. Zhu Y. Romain C. Brooks R. Saini P. K. Williams C. K. (2015). Ring-opening copolymerization (ROCOP): synthesis and properties of polyesters and polycarbonates. Chem. Commun. 51, 6459–6479. 10.1039/C4CC10113H 25688813

[B73] Penczek S. Kubisa P. Matyjaszewski K. (1980). Monomer structures, ring strains and nucleophilicities (Basicities), in Cationic Ring-Opening Polymerization of Heterocyclic Monomers, eds Penczek S. Kubisa P. Matyjaszewski K. (Berlin; Heidelberg: Springer), 3–7.

[B74] Piedra-Arroni E. Ladavière C. Amgoune A. Bourissou D. (2013). Ring-opening polymerization with Zn(C6F5)2-based lewis pairs: original and efficient approach to cyclic polyesters. J. Am. Chem. Soc. 135, 13306–13309. 10.1021/ja4069968 23987101

[B75] Pounder R. J. Dove A. P. (2010). Synthesis and organocatalytic ring-opening polymerization of cyclic esters derived from l-Malic Acid. Biomacromolecules 11, 1930–1939. 10.1021/bm1004355 20690706

[B76] Pounder R. J. Fox D. J. Barker I. A. Bennison M. J. Dove A. P. (2011). Ring-opening polymerization of an O-carboxyanhydride monomer derived from L-malic acid. Polym. Chem. 2, 2204–2212. 10.1039/c1py00254f

[B77] Prier C. K. Rankic D. A. MacMillan D. W. C. (2013). Visible light photoredox catalysis with transition metal complexes: applications in organic synthesis. Chem. Rev. 113, 5322–5363. 10.1021/cr300503r 23509883 PMC4028850

[B78] Rahimi A. García J. M. (2017). Chemical recycling of waste plastics for new materials production. Nat. Rev. Chem. 1:0046 10.1038/s41570-017-0046

[B79] Rasal R. M. Janorkar A. V. Hirt D. E. (2010). Poly(lactic acid) modifications. Prog. Polym. Sci. 35, 338–356. 10.1016/j.progpolymsci.2009.12.003

[B80] Saiyasombat W. Molloy R. Nicholson T. M. Johnson A. F. Ward I. M. Poshyachinda S. (1998). Ring strain and polymerizability of cyclic esters. Polymer 39, 5581–5585. 10.1016/S0032-3861(97)10370-6

[B81] Sano K. Yamamoto T. Yamamoto A. (1984). Preparation of Ni-containing or Pt-containing cyclic esters by oxidative addition of cyclic carboxylic anhydrides and their properties. Bull. Chem. Soc. Jpn. 57, 2741–2747. 10.1246/bcsj.57.2741

[B82] Sardon H. Dove A. P. (2018). Plastics recycling with a difference. Science 360, 380–381. 10.1126/science.aat4997 29700253

[B83] Schneiderman D. K. Hillmyer M. A. (2017). 50th anniversary perspective: there is a great future in sustainable polymers. Macromolecules 50, 3733–3750. 10.1021/acs.macromol.7b00293

[B84] Slomkowski S. Penczek S. Duda A. (2014). Polylactides—an overview. Polym. Adv. Technol. 25, 436–447. 10.1002/pat.3281

[B85] Smith I. J. Tighe B. J. (1976). Studies in ring-opening polymerization. 4. Thermal polymerization of phenyl substituted 1,3-dioxolan-2,4-diones. J. Polym. Sci. Polym. Chem. 14, 949-960. 10.1002/pol.1976.170140415

[B86] Smith I. J. Tighe B. J. (1981). Studies in ring-opening polymerization. 6. Tertiary base initated polymerization of 5-phenyl-1,3-dioxolan-2,4-dione. Macromol. Chem. Phys. 182, 313-324. 10.1002/macp.1981.021820204

[B87] Södergård A. Stolt M. (2002). Properties of lactic acid based polymers and their correlation with composition. Prog. Polym. Sci. 27, 1123–1163. 10.1016/S0079-6700(02)00012-6

[B88] Stanford M. J. Dove A. P. (2010). Stereocontrolled ring-opening polymerisation of lactide. Chem. Soc. Rev. 39, 486–494. 10.1039/B815104K 20111773

[B89] Sun Y. Jia Z. Chen C. Cong Y. Mao X. Wu J. (2017). Alternating sequence controlled copolymer synthesis of α-Hydroxy acids via syndioselective ring-opening polymerization of O-Carboxyanhydrides using zirconium/hafnium alkoxide initiators. J. Am. Chem. Soc. 139, 10723–10732. 10.1021/jacs.7b04712 28715211

[B90] Szwarc M. (1956). ‘Living’ Polymers. Nature 178:1168.

[B91] Tang L. Deng L. (2002). Dynamic kinetic resolution via dual-function catalysis of modified cinchona alkaloids: asymmetric synthesis of α-Hydroxy carboxylic acids. J. Am. Chem. Soc. 124, 2870–2871. 10.1021/ja0255047 11902867

[B92] Thomas C. M. (2010). Stereocontrolled ring-opening polymerization of cyclic esters: synthesis of new polyester microstructures. Chem. Soc. Rev. 39, 165–173. 10.1039/B810065A 20023847

[B93] Tong R. (2017). New chemistry in functional aliphatic polyesters. Ind. Eng. Chem. Res. 56, 4207–4219. 10.1021/acs.iecr.7b00524

[B94] Toyooka K. Takeuchi Y. Kubota S. (1989). A noval and facile synthesis of 5-substituted 1,3-dioxolan-2,4-diones using trichloromethyl chloroformate. Heterocycles 29, 975–978. 10.3987/COM-89-4939

[B95] Van de Velde K. Kiekens P. (2002). Biopolymers: overview of several properties and consequences on their applications. Polym. Test. 21, 433–442. 10.1016/S0142-9418(01)00107-6

[B96] Vandenbossche C. P. de Croos P. Singh S. P. Bakale R. P. Wagler T. R. (2010). Formation of (S)-5-Cyclohexyl-5-phenyl-1,3-dioxolane-2,4-dione: a key intermediate in the synthesis of (S)-Oxybutynin Hydrochloride. Org. Process Res. Dev. 14, 921–925. 10.1021/op100021w

[B97] Wang R. Zhang J. Yin Q. Xu Y. Cheng J. Tong R. (2016). Controlled ring-opening polymerization of O-Carboxyanhydrides using a β-Diiminate Zinc Catalyst. Angew. Chem. Int. Chem. 55, 13010–13014. 10.1002/anie.201605508 27634170

[B98] Xia H. D. Kan S. L. Li Z. J. Chen J. Cui S. D. Wu W. Z. (2014). N-Heterocyclic carbenes as organocatalysts in controlled/living ring-opening polymerization of O-Carboxyanhydrides derived from L-Lactic Acid and L-Mandelic Acid. J. Polym. Sci. Polym. Chem. 52, 2306–2315. 10.1002/pola.27241

[B99] Yamamoto T. Ishizu J. Kohara T. Komiya S. Yamamoto A. (1980). Oxidative addition of aryl carboxylates to Ni(0) complexes involving cleavage of the aryl-O bond. J. Am. Chem. Soc. 102, 3758–3764. 10.1021/ja00531a016

[B100] Yin Q. Tong R. Xu Y. Baek K. Dobrucki L. W. Fan T. M.. (2013). Drug-initiated ring-opening polymerization of O-Carboxyanhydrides for the preparation of anticancer drug-Poly(O-carboxyanhydride) nanoconjugates. Biomacromolecules 14, 920–929. 10.1021/bm301999c 23445497 PMC3671392

[B101] Yin Q. Yin L. Wang H. Cheng J. (2015). Synthesis and biomedical applications of functional Poly(α-hydroxy acids) via ring-opening polymerization of O-Carboxyanhydrides. Acc. Chem. Res. 48, 1777–1787. 10.1021/ar500455z 26065588

[B102] Yu Y. Zou J. Cheng C. (2014). Synthesis and biomedical applications of functional poly([small alpha]-hydroxyl acid)s. Polym. Chem. 5, 5854–5872. 10.1039/C4PY00667D

[B103] Zhang L. Nederberg F. Messman J. M. Pratt R. C. Hedrick J. L. Wade C. G. (2007). Organocatalytic stereoselective ring-opening polymerization of lactide with dimeric phosphazene bases. J. Am. Chem. Soc. 129, 12610–12611. 10.1021/ja074131c 17900113

[B104] Zhang Z. Yin L. Xu Y. Tong R. Lu Y. Ren J.. (2012). Facile functionalization of polyesters through thiol-yne chemistry for the design of degradable, cell-penetrating and gene delivery dual-functional agents. Biomacromolecules 13, 3456–3462. 10.1021/bm301333w 23098261 PMC3606898

[B105] Zhu J.-B. Chen E. Y. X. (2015). From meso-Lactide to isotactic polylactide: epimerization by B/N lewis pairs and kinetic resolution by organic catalysts. J. Am. Chem. Soc. 137, 12506–12509. 10.1021/jacs.5b08658 26388298

[B106] Zhu J.-B. Watson E. M. Tang J. Chen E. Y.-X. (2018). A synthetic polymer system with repeatable chemical recyclability. Science 360, 398–403. 10.1126/science.aar5498 29700260

[B107] Zhu Y. Romain C. Williams C. K. (2016). Sustainable polymers from renewable resources. Nature 540, 354–362. 10.1038/nature21001 27974763

[B108] Zhuang X.-l. Yu H.-y. Tang Z.-h. Oyaizu K. Nishide H. Chen X.-s. (2010). Polymerization of lactic O-carboxylic anhydride using organometallic catalysts. Chin. J. Polym. Sci. 29, 197–202. 10.1007/s10118-010-1013-7

[B109] Zuo Z. W. Ahneman D. T. Chu L. L. Terrett J. A. Doyle A. G. MacMillan D. W. C. (2014). Merging photoredox with nickel catalysis: coupling of alpha-carboxyl sp-carbons with aryl halides. Science 345, 437–440. 10.1126/science.1255525 24903563 PMC4296524

